# Influence of Konjac Glucomannan and Frozen Storage on Rheological and Tensile Properties of Frozen Dough

**DOI:** 10.3390/polym11050794

**Published:** 2019-05-02

**Authors:** Tingting Cui, Rui Liu, Tao Wu, Wenjie Sui, Min Zhang

**Affiliations:** 1 State Key Laboratory of Food Nutrition and Safety, Tianjin University of Science & Technology, Tianjin 300457, China; ctt1519@163.com (T.C.); lr@tust.edu.cn (R.L.); wutao@tust.edu.cn (T.W.); wjsui@tust.edu.cn (W.S.); 2 Key Laboratory of Food Nutrition and Safety, Tianjin University of Science & Technology, Tianjin 300457, China; 3 Engineering Research Center of Food Biotechnology, Ministry of Education, Tianjin 300457, China

**Keywords:** konjac glucomannan, gluten, frozen dough, interactions, physicochemical properties

## Abstract

The impact of various amounts of konjac glucomannan on the structural and physicochemical properties of gluten proteins/dough at different periods of frozen storage is evaluated in the present study. As frozen storage time was prolonged, the molecular weight and the free sulfhydryl content of gluten proteins and the tensile properties of frozen dough all decreased. The addition of konjac glucomannan reduced the variations in the structural and rheological properties of gluten proteins/dough. Frozen dough with 2.5% added konjac glucomannan showed the highest water binding capacity and retarded the migration of water. Scanning electron microscopy and differential scanning calorimetry results also revealed that adding konjac glucomannan reduced the cracks and holes in the dough and enhanced its thermal stability. The correlations between mechanical characteristics and structure parameters further indicated that konjac glucomannan could not only stabilize the structures of gluten proteins but also bind free water to form more stable complexes, thereby retaining the rheological and tensile properties of the frozen dough.

## 1. Introduction

Frozen dough is increasingly used in making baked food and more Chinese cuisine, such as sweet dumpling balls, steamed buns, and dumplings [1], because of its advantages in many perspectives, such as saving time and retail expenses, prolonging the shelf-life of dough and facilitating standardization and large-scale production [2,3]. In recent years, freezing technology has been intensively studied and rapidly developed for preserving dough. However, there are several problems in producing baking food from frozen dough. For instance, the frozen dough often has poor gas retention, shrunk bread volume, loss of flavor, disintegrated crumb structure, and deterioration in the texture of final products. The overall quality of frozen dough declined gradually in frozen storage [4], which is closely related to the formation and change of the three-dimensional viscoelastic dough network. It depends on the intermolecular crosslinking of wheat gluten proteins and a variety of factors that can affect the crosslinking. For example, Virginia and Tzia [5] suggested that the water redistribution in frozen dough became uneven during freeze-thaw cycles, which was likely to degrade the quality of dough. Temperature fluctuations during storage and transportation could also decrease the dough’s quality by recrystallization [6]. Therefore, controlling the freezing rate and the temperature stability is essential to protect the structure and properties of the gluten network and the dough’s quality.

Besides optimizing the freezing rate and avoiding temperature change, additives, especially hydrocolloids, are a practical way to increase the rheological and thermophysical properties of frozen dough [7]. Hydrocolloids can protect the structure and rheological properties of frozen dough during freeze-thaw cycles. It can combine with gluten and bound water to form a complex, thereby reducing the migration of moisture [8]. Hydrocolloids can also decrease water activity because they compete with proteins and starches to bind water. In addition, Xuan et al. [9] demonstrated that hydroxypropyl methylcellulose could avoid the recrystallization of water to stabilize the microstructure and conformation of the gluten network. To protect the structure and properties of frozen dough, the utilization of hydrocolloids is an effective way to stabilize the quality of frozen dough and its final foods [10].

Konjac glucomannan (KGM), as a high molecular weight polysaccharide, is extracted from the tuberous roots of konjac. As a neutral polysaccharide, KGM is composed of a linear chain of *β*-1,4-linked D-glucose, and D-mannose residues at a molar ratio of 1:1.6 [11]. Due to its good rheological properties, KGM is often used as an additive and thickener to improve textural, sensory, rheological, and microstructural properties of dough products and many other materials [12,13,14]. As far as hydrocolloids incorporated in frozen dough was concerned, previous studies have reported carboxymethyl cellulose (CMC), κ-carrageenan, arabic gum, locust bean gum, etc. to improve the quality of frozen dough. However, to the best of our knowledge, there is little information on the application of KGM in frozen dough, the effects of KGM on the microstructural and physicochemical properties of gluten proteins in frozen dough, or its protective mechanism on dough quality.

Therefore, the objective of this study was the impact of KGM on the structural and physicochemical variation of gluten proteins to probe into the relationship between KGM and gluten proteins in frozen dough and to explore the mechanism of protective effect of KGM for frozen dough. In particular, correlations between mechanical characteristics and structure parameters were also determined to gain more insights about the protective effect of KGM for frozen dough. This study will provide further evidence for exploring the interactions between hydrocolloids and gluten proteins and, thus, benefit the production and application of frozen dough.

## 2. Materials and Methods

### 2.1. Materials

Wheat flour made by 14.38% protein, 72.19% starch, 0.45% ash, and 12.83% moisture (dry basis) was supplied by the Henan Wudeli Flour Group Corp, Henan, China. Protein, starch, ash, and moisture content analyses of wheat flour were performed according to American Association of Cereal Chemists (AACC) [15]. Konjac glucomannan (KGM) ≥98% purity was provided by the Hefei Bomei Biotechnology Corp, China. All other reagents and chemicals were of analytical purity. Deionized distilled water was used for all experiments.

### 2.2. Dough Preparation

Three grams of KGM was mixed with 100 mL of deionized distilled water. The mixture was swollen for 30 min to form a transparent homogeneous solution. A total of 50 g of wheat flour in a KGM (0.0%, 0.5%, 1.0%, 1.5%, 2.0%, and 2.5% of wheat flour dry basis) solution and deionized distilled water were mixed and kneaded for 5 min, while the sample without KGM was used as the control group. The total amount of deionized distilled water was 55% of the wheat flour at a dry basis. Dough was covered with plastic wrap in food packaging and fermented for approximately 1 hour until its size was doubled. The dough was quickly frozen in liquid nitrogen and stored at −18 °C for 15, 30, 45, and 60 days, and dough pieces were thawed at 25 °C. Then, dough was washed in a 2.0% sodium chloride solution to prepare gluten proteins and was lyophilized for further analyses.

### 2.3. Molecular Weight (Mw) Analysis of Gluten Proteins

Size-exclusion high performance liquid chromatography (SE-HPLC) measurement was carried on a Shimadzu LC-20AT HPLC system equipped with a RF-20A UV-vis Detector (Tokyo, Japan) according to the method of Chaudhary et al. [16]. Gluten proteins with different proportions of KGM, added for different frozen periods, were extracted by adding 50 mg of the freeze-dried gluten protein samples to 1 mL of an acetic acid solution (500 mM). The insoluble part that accounted for about 20% of the total gluten protein samples was removed by centrifugation for 10 min at 5000 r/min. The soluble part of gluten protein sample was filtered through a 0.22 μm PVDF membrane filter and then subjected to SE-HPLC analysis. A 20 μL gluten protein sample was injected into a size-exclusion column (Biosep-SEC-S4000, Phenomenex, 300 × 7.8 mm, Torrance, CA, USA). The samples were eluted with an acetic acid solution (500 mM) at a flow rate of 0.8 mL/min and were detected at 280 nm. A calibration curve with *r*^2^ > 0.9 was obtained by plotting the peak area of the ribonuclease (*Mw* 1.37 × 10^5^ Da), ovalbumin (*Mw* 4.43 × 10^5^ Da), γ-globulin (*Mw* 1.50 × 10^5^ Da), and bovine thyroglobulin (*Mw* 6.70 × 10^5^ Da), all of which were obtained from Sigma Chemical Co. (St. Louis, MO, USA).

### 2.4. Free Sulfhydryl Content Analysis of Gluten Proteins

The free sulfhydryl group (*SH*_F_) content of gluten proteins with different proportions of KGM at different frozen storage times was determined by following the method described by Beveridge et al. [17]. Freeze-dried wheat gluten (5 mg) was dissolved in 5 mL of a urea solution (8 mol/L, 1.04% Tris, 1 mM EDTA, 0.69% Gly, 1.5% SDS, 8 M Urea, pH 8.0) and was centrifuged at 3000 r/min for 10 min. Then, 1 mL of the suspension was mixed with 2 mL of a Tris-Gly solution (1.04% Tris, 1 mM EDTA, 0.69% Gly, 1.5% SDS) and 200 μL of DTNB reagent (4 mg/mL), and the mixture was shaken for 30 min at room temperature. The absorbance of each supernatant was 412 nm. To measure the total sulfhydryl equivalent groups (*SH*_eq_), 2 mL of a Tris-Gly solution and 0.02 mL *β*-mercaptoethanol were continuously added to 1 mL of supernatant after being centrifuged and shaken for 1 h at room temperature. Then, 10 mL of 12% trichloroacetic acid was added to precipitate the gluten proteins. The sediment was washed with 12% trichloroacetic acid and centrifuged three times at 3000 r/min for 10 min. The gluten protein sediment was re-dissolved in 10 mL of a Tris-Gly solution and 0.04 mL of DTNB reagent. The absorbance was recorded at 412 nm. The *SH*_F_ and *SH*_eq_ values were calculated using the following equation [17]:

*SH*_F_ (μmoL/g) = 73.53 × *A*_412_ × *D*/*C*, (1)

where 73.53 = 10^6^/(1.36 × 10^4^) and 1.36 × 10^4^ are molar absorption coefficients of DTNB, *A*_412_ is the absorbance of the sample at 412 nm, *D* is the dilution factor of the sample, and *C* is the sample concentration (mg/mL). In addition, the disulfide bond (*SS*) content was calculated from *SH*_F_ and *SH*_eq_ [17], as follows:

*SS* = (*SH*_eq_ − *SH*_F_)/2. (2)

### 2.5. Secondary Structure Analysis of Gluten Proteins

The study on secondary structure of gluten proteins with different proportions of KGM at different frozen storage times was operated with Fourier transform infrared (FTIR) spectroscopy. FTIR spectra were recorded over the wavelength range of 4000 cm^−1^ to 400 cm^−1^ using a Nicolet IS50 FTIR spectrometer (Thermo Nicolet Corp, Madison, WI, USA) equipped with a single-reflection diamond attenuated total reflection (ATR) crystal and a mercury-cadmium-telluride (MCT) detector. A total of 1.0 mg of gluten protein sample was mixed with 150 mg KBr powder and compressed into discs at a force of 5 KN for 30 s. FTIR spectra were recorded with 64 scans and a 4 cm^−1^ resolution against the background. The secondary structure of the samples was analyzed by the OMNIC software package (version 8.0, Thermo Nicolet Corp, Madison, WI, USA) and Origin software (version 9.1).

### 2.6. Water Fluidity Analysis of Dough

Adding different amounts of KGM at different frozen storage times (thawed at 25 °C), the water fluidity of dough was measured by low-frequency nuclear magnetic resonance (LF-NMR) to analyze *T*_2_, according to Xuan et al. [9]. The NMR probe (10 mm diameter) was filled with 2.0–3.0 g of dough and hermetically sealed with a plastic wrap. Transverse relaxation curves were calculated by a Carr-Purcell-Meiboom-Gill (CPMG) pulse sequence. The resonance frequency was set at 22 Hz, magnetic strength was at 0.5 T, coil diameter was at 60 mm, magnetic temperature was at 32 °C, and the lengths of the 90° and 180° pulses were at 14 ms and 35 ms, respectively. For the measurements, a recycle delay of 1 s was adopted and 8 scans were accumulated to increase the signal-to-noise ratio (SNR). A *T*_2_ distribution curve was calculated by the following equation [9]:

(3) $$M\left(t\right)\;=\;\int_{0}^{\infty }F\left(T\right)\exp\left(-\frac{t}{T}\right)dT,$$

where M is a sum of exponential decays of signal amplitude as a function of time (*t*) and F(*T*) is the number density of protons as a function of relaxation time (*T*).

The CONTIN algorithm from Provencher software (Newmai, SuZhou, China) was used to transform the transverse relaxation curves with an inverse Laplace transformation to continuous distributions of *T*_2_ values.

### 2.7. Thermal Stability Analysis of Gluten Proteins

The thermal transition patterns of freeze-dried samples were analyzed by a DSC system (DSC-60 Plus, Shimadzu, Corp, Kyoto, Japan) with a computerized data station (TA-60 WS), according to Yang et al. [18]. Gluten proteins were freeze-dried and ground through a 120-mesh sieve. The sample, measuring 3.0–5.0 mg, was sealed into an aluminum pan (S201-52943, Shimadzu, Japan) and equilibrated at 30 °C for 5 min, followed by being heated (up to 250 °C) with constant nitrogen-purging at a constant rate of 5 °C/min. A sealed empty aluminum pan was used as a reference. The enthalpy change (Δ*H*) and peak temperature (*T*_p_) of gluten proteins with different amounts of KGM at different frozen storage times were determined by the TA-60 Analysis software (version 2.21, Shimadzu Corp, Japan).

### 2.8. Microstructure of Dough

The microstructures of various dough samples were observed by scanning electron microscopy (SEM) based on the method of Huang et al. [19]. Freeze-dried dough samples were coated by gold particles in a sputter coater. Images were taken by a SU-1510 scan electron microscope (Hitachi Corp, Mito, Japan) with a 5 KV acceleration voltage at a magnification of 500×.

### 2.9. Rheological Properties of Dough

Rheological property experiments were operated in a controlled-stress HAAKE MARS-III rheometer (Thermo Scientific Corp, Waltham, Germany), according to the method of Wu et al. [20]. The frozen dough was thawed at room temperature in a 20 mm diameter steel plate, gapped by 1 mm. A thin film of methyl silicone oil was gently applied at the edge of the steel plate to prevent moisture loss. A frequency sweep was conducted from 0.1 to 100 rad/s for each sample, using a constant strain of 1.0% at 25 °C. The storage modulus (*G′*) and loss modulus (*G″*) were recorded and the loss factor (*tanδ*) was reported as *G″*/*G′*.

### 2.10. Tensile Tests of Dough

Dough stored for different frozen periods and thawed at 25 °C was studied using samples with dimensions of 30 × 30 × 30 mm. The tensile test was operated by TA.XT. Plus texture analyzer with a Kieffer extensibility rig. Strips of dough (Φ1 mm × 50 mm) were mounted in tensile grips at a 5.0 mm/s pre-test speed, 2.0 mm/s test speed, 5.0 mm/s post-test speed, with 5.0 s test time, 0.05 N trigger force, and 75.0 mm distance. The *R_k_^max^* (maximum resistance), *E^k^* (extensibility), and *A^k^* (extension area, estimated by calculating *R_k_^max^* × *E^k^*) and stretch ratio were calculated by *R_k_^max^* (maximum resistance) and *E^k^* (extensibility).

### 2.11. Statistical Analysis

Statistical data analysis of three independent replicates were performed and the data are expressed as the mean ± standard deviation values. The results were calculated and graphs were obtained using Origin software (version 9.1), followed by one-way analysis of variance (ANOVA) at a significance level of 0.05.

## 3. Results and Discussion

### 3.1. Structural Properties of Gluten Proteins

#### 3.1.1. *M*_w_ Analysis of Gluten Proteins

**Figure 1:**
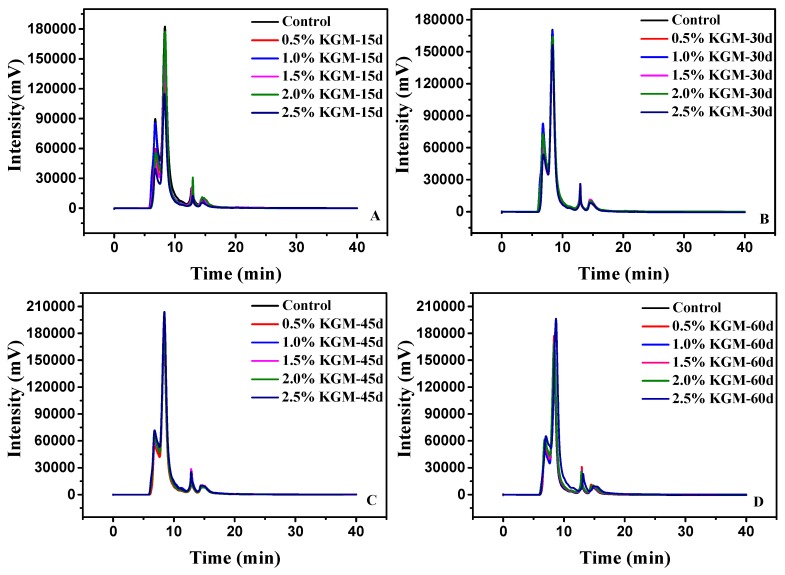
Size-exclusion HPLC chromatogram of gluten proteins in frozen dough with different proportions of KGM added. 15 days frozen storage (**A**), 30 days frozen storage (**B**), 45 days frozen storage (**C**), and 60 days frozen storage (**D**).

Figure 1 shows that gluten proteins were divided into four fractions, which was consistent with the result of Manu et al. [21]. According to Manu et al. [21], Peaks 1–4 represented high-molecular weight glutenin polymers (Peak 1, *M*_w_ = 3.70 × 10^5^ Da–6.88 × 10^5^ Da ), low-molecular weight glutenin polymers (Peak 2, *M*_w_ = 9.10 × 10^5^ Da–3.70 × 10^5^ Da), gliadins (Peak 3, *M*_w_ = 1.60 × 10^4^ Da–9.10 × 10^4^ Da), and other low-molecular weight peptides or phenolic compounds (Peak 4, *M*_w_ < 1.0 × 10^4^ Da), respectively. The *M*_w_ and relative percentage of different fractions were calculated and shown in Table 1. With longer frozen storage times, the retention times of Peak 1 and Peak 2 (Figure 1) for the frozen samples were negatively delayed and the areas decreased, compared with those of the fresh sample, which indicated that the *M*_w_ represented by Peak 1 and Peak 2 decreased. For samples with 2.5% KGM after being frozen 60 days, the Peak 1 and Peak 2 areas decreased from 29.24% and 51.83% to 23.24% and 44.39%, respectively. The protein loss might be caused by the depolymerization of gluten polymeric proteins as a result of ice recrystallization and water redistribution in frozen storage [4]. Zhao et al. [22] further demonstrated that molecular weight ranging from 3.0 × 10^5^ Da–4.0 × 10^8^ Da decreased while frozen storage time increased under freeze-thaw conditions and the molecular weight of gluten decreased due to the breakage of intermolecular disulfide bonds between gluten polymers.

However, there was a small change in the retention time of Peak 3 and its area increased slightly. Peak 4 increased more than Peak 3, indicating that monomeric proteins are evidence of the depolymerization of gluten polymeric proteins during frozen storage. The damage to gluten polymers increased for the sample stored for 60 days, surpassing that of other samples.

Added with KGM, Peak 1 and Peak 2 increased from 23.35% and 48.59% to 29.24% and 51.83% for fresh dough, respectively (Table 1). For each sample with different frozen storage times, Peak 1 and Peak 2 also increased, indicating that dough samples with KGM were resistant to the depolymerization effect. Large and medium glutenin polymers were found to make the greatest contribution to dough properties. Their amount and properties were found to be closely related to the dough strength and loaf volume [23]. Moreover, adding KGM to wheat flour can primarily affect the textural properties of hardness and springiness with a reinforcing gluten network [24]. From this perspective, KGM could inhibit gluten protein depolymerization and maintain the network structure stability.

#### 3.1.2. Secondary Structural Contents of Gluten Proteins

The secondary structure of gluten proteins with different proportions of KGM for different frozen storage time was studied using the FTIR and spectra, as exhibited in Figure S1.

**Figure 2:**
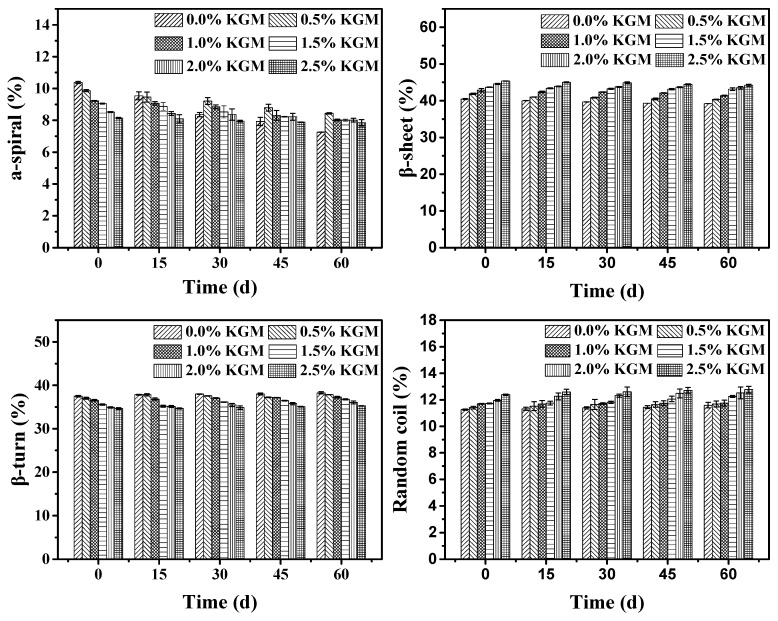
Secondary structure changes of gluten proteins with different proportions of KGM added during frozen storage.

The amide I (1600–1700 cm^−1^) band was used to analyze the secondary structure of gluten proteins due to its high sensitivity and strong intensity, which was attributed to intermolecular *α*-helices (1650–1660 cm^−1^), *β*-sheet (1612–1640 cm^−1^), *β*-turn (1662–1670 cm^−1^), and random coil (1642–1648 cm^−1^) structures [25]. Quantitative estimations of different structure fractions of gluten proteins are shown in Figure 2. The *β*-sheet was significantly influenced by the different proportions of KGM. As the amount of KGM increased, the *β*-sheet increased from 40.45% to 45.37%. As the frozen storage time increased, the *β*-sheet decreased from 40.45% to 39.22%. At the same time, the *β*-turn content increased with KGM addition. Although *β*-sheets and *β*-turns interconvert during this process, *β*-turn increased with the increased frozen storage time, because the depolymerization and structure of gluten proteins were destroyed by ice crystals. This result was in accordance with the results of the rheological analysis and changes of the free sulfhydryl group. A significant decrease was observed in *α*-helices and the *β*-turn during frozen storage, while the *β*-sheet and random coils decreased. In summary, the freeze-thaw cycle stability and water retention of frozen dough or flour products are of vital importance to its quality and, thus, frozen dough requires a much stronger gluten network than ordinary dough.

#### 3.1.3. Free Sulfhydryl Content of Gluten Proteins

**Figure 3:**
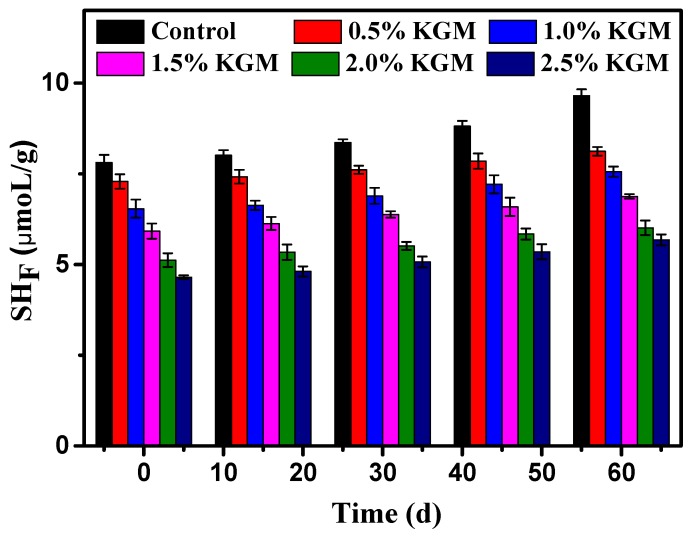
Free sulfhydryl content (E) of gluten proteins with different proportions of KGM added during frozen storage.

Disulfide bonds play an important role in maintaining the structural stability of gluten. Glutenin is a polymer class linked by intra/intermolecular disulfide bonds, while gliadin is a mixture composed of single chains with largely intramolecular disulfide bonds [26]. Changes of the free sulfhydryl are an important indicator of modifications to disulfide bonds. As shown in Figure 3, the free sulfhydryl increased in all of the frozen samples, along with disulfide bond breakage caused by ice crystallization. This result is in accordance with the SE-HPLC results and is related to the depolymerization of gluten polymeric proteins, since smaller glutenin molecules are connected by inter-disulfide bonds to form polymeric proteins. Therefore, the breakage of inter-disulfide bonds would directly decrease the polymeric protein content [27]. Additionally, ice crystal formation and water migration were regarded as the main contributors to this phenomenon.

Figure 3 shows that the free sulfhydryl increased as the freeze time increased. The free sulfhydryl of fresh samples was 7.81 μmol/g; while with a frozen time up to 60 days, the free sulfhydryl of fresh samples increased to 9.65 μmol/g, because ice crystals destroyed the gluten structure during the freeze-thaw process. Zhao et al. [22] found that high molecular weight gluten proteins were depolymerized, mainly between 10^5^ Da–10^9^ Da, due to internal gluten proteins disulfide bond rupture. Freeze-thaw cycles lead to the depolymerization of gluten proteins, which mainly center at approximately 3 × 10^5^ Da–4 × 10^8^ Da. Additionally, dead yeast releases glutathione, which acts as a kind of reductant that can directly or indirectly break disulfide bonds between gluten proteins and prevent CO_2_ retention, which has impact on the three-dimensional network structure of gluten and the rheological properties of dough [27].

With KGM, the sulfhydryl decreased for every group. In fresh samples, sulfhydryl decreased from 7.81 μmol/g to 4.65 μmol/g and the sulfhydryl transformed into a disulfide bond. When the frozen storage time was extended to 60 days, the sulfhydryl decreased from 9.65 μmol/g to 5.68 μmol/g. This decrease was larger than that in fresh group because KGM has good water adsorption ability. Although the freeze-thaw cycle process may produce many ice crystals and the water distribution will change, the damage to dough can be avoided by adding KGM. Since glutenin is a polymeric protein formed by the polymerization of multiple subunits through disulfide bonds outside the chain, it can contribute to the strength and elasticity of dough [28]. Disulfide bonds were protected by KGM and have influence on dough’s quality, as the result of the *T*_2_ relaxation time showed.

### 3.2. Interactions Between KGM and Gluten Proteins

#### 3.2.1. T_2_ distributions of Frozen Dough

**Figure 4:**
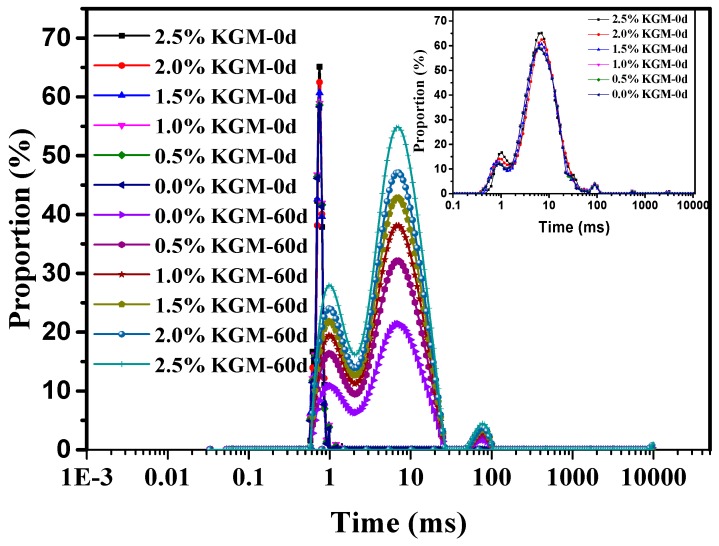
*T*_2_ distribution curve of dough with different proportions of KGM added during frozen storage.

The water binding capacity and fluidity plays an important role in food, since they have direct effects on the rheological properties and stability of the final products [29]. Freezing is a popular method to process and store food and the distribution of water is often changed during this process, giving rise to a series of physical and chemical changes that are all related to the properties of dough, such as protein degeneration and enzymatic activities. Freezing is also deemed to be an irreplaceable determinant of food rheology. Therefore, it is necessary to study the change of water in food during frozen processes. The *T*_2_ relaxation time is an important parameter of LF-NMR technology, which can reveal the fluidity of water in complex food systems. The *T*_2_ distribution curve is shown in Figure 4. The values *T*_21,_ *T*_22_, and *T*_23_ represent bound water, immobilized water, and free water, respectively [30], while the peak area proportions of water in frozen dough with different additive amounts of KGM were calculated and are recorded in Table 2.

The *T*_21_ and *T*_22_ values significantly increased with the increase of KGM. KGM can combine with significant amounts of water through hydrogen bonding, molecular dipoles, and macromolecules, which are hard to move. It was reported that KGM has high water absorbency, near 105.4 g/g (water/KGM) [31]. After the addition of KGM, intra-granular water in gluten is absorbed by KGM in a certain spatial structure and reduces water fluidity [32].

With the addition of 2.5% KGM, *T*_21_ increased from 9.20% to 10.09% after a frozen storage time of 60 days; *T*_22_ increased from 77.65% to 80.96%, and *T*_23_ decreased from 13.15% to 8.95%. At the same frozen storage time and adding the same amount of KGM, the increase in the amplitude of *T*_22_ was larger than that of *T*_21_, which demonstrated that immobilized water was the principal water component of dough and that the combination of gluten and water led to increased tightness. In general, it could be inferred as KGM weakened the influence of frozen storage treatment on water mobility and it had a positive effect on dough quality during storage, which led to the status of water shifting from *T*_23_ to *T*_22_ and *T*_21_ in frozen storage. As the frozen storage time increases, ice formation will destroy the structure of the gluten network, increase free water, and significantly decrease the amount of immobilized water. For example, for dough samples without KGM and a freeze time from 0 to 60 days, the *T*_21_ increased from 0.37% to 1.98%, while *T*_22_ decreased from 80.05% to 77.65% and *T*_23_ increased from 9.19% to 13.15%.

Moreover, reduction of the *T*_22_ area reflects decreased water availability in the gluten system, which reduces gluten strength. The gluten homogeneous structure state was broken for water migration during frozen storage. By contrast, after the addition of KGM, the mechanical properties of dough samples were superior to those of control group samples, suggesting that KGM showed better beneficial effects for inhibiting the change of freezable water and made the water distribution more uniform. This phenomenon can be explained by thermal stability analysis and is consistent with the resultant free sulfhydryl content.

#### 3.2.2. Thermal Stability of Frozen Dough

Ice crystals formed in frozen storage can destroy the gluten structure and have a direct impact on the properties and function of protein. Gluten degeneration is associated with enthalpy changes. DSC can provide information, such as the effects of molecular interactions on protein denaturation and processing conditions on protein functional properties. According to the endothermic processes displayed in the DSC thermogram, information about the structural stability and thermal effects on protein configuration changes can be determined. Space alterations during thermal denaturation and protein conformation changes may occur, such as chain stretches and folds, which can cause chemical group restructuring and thermal deformation due to protein physiological activity changes [33].

According to the DSC spectra (Figure S2), the temperature and peak area corresponding to the peak point can be used to determine the temperature variability enthalpy of this transformation. The effect of the protein denaturation temperature on thermal stability and enthalpy changes of hydrophobic or hydrophilic protein molecules represent the energy of this reaction and the peak width explains the collaborative degeneration of molecules. If a change occurs in a very narrow temperature range, it indicates that the reaction is strongly collaborative. As seen from Table 2, the denaturation temperature (*T*_p_) of fresh dough samples reached its peak value at a frozen storage time of 60 days. Without KGM, these values were 54.74 °C and 62.62 °C, respectively. For each group, *T*_p_ increased with longer frozen storage time, in general agreement with data reported by Wang et al. [34]. The *T*_p_ of the control group and dough with KGM increased by 7.88 °C and 10.48 °C, respectively, after 60 days in frozen storage. High *T*_p_ values are expected in proteins with a high proportion of hydrophobic residues involved in the denaturation mechanism [2]. Therefore, the high *T*_p_ values obtained in this study were probably due to the high hydrophobicity of the gluten fractions.

With different proportions of KGM, for all samples, the enthalpy (∆*H*) decreased with the frozen storage time, suggesting that gluten proteins underwent progressive denaturation during freezing and subsequent frozen storage involving a disruption of the ordered structure. The disrupted ordered structures of gluten proteins can be interpreted as deterioration, which could weaken the properties of the final baked products [2]. This result was in accordance with the *M*_w_, disulfide bond, and secondary structure changes of gluten proteins and these thermal stability results can explain the deterioration of the rheological and tensile properties.

#### 3.2.3. Microstructure of Frozen Dough

**Figure 5:**
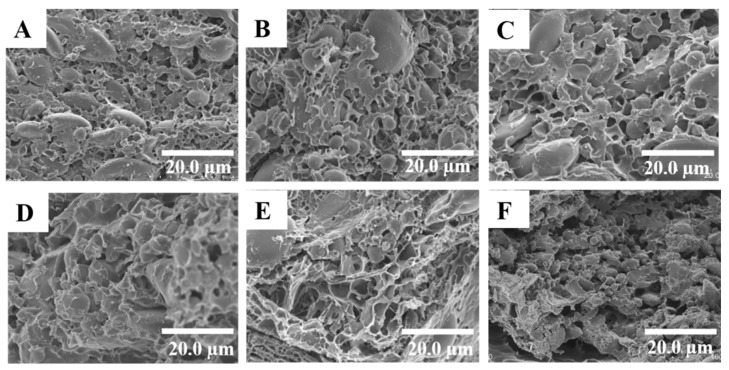
Scanning electron micrographs of frozen dough with different proportions of KGM added during frozen storage. Fresh dough sample with 2.5% KGM (**A**), frozen for 60 days with 2.5% KGM (**B**), frozen for 60 days with 2.0% KGM (**C**), frozen for 60 days with 1.5% KGM (**D**), frozen for 60 days with 1.0% KGM (**E**), and frozen for 60 days with 0.5% KGM (**F**).

The effect of the proportion of KGM added on the microstructure of frozen dough is shown in Figure 5. Significant differences in the microstructure among different dough samples were observed in these images. Figure 5A shows that the control group has the tightest and most uniform network. However, this continuous and uniform gluten network (Figure 5B–F) was destroyed by freezing. The gluten system appeared to be less continuous, more disrupted, and more separated from the starch granules. The most obvious sample is shown in Figure 5E, where more holes in the dough were apparent with no KGM addition and the frozen storage time was up to 60 days. This phenomenon was in agreement with trends observed by Luo et al. [35]. These image results were consistent with the results from the examination of the secondary structure and thermal properties obtained in this study, which implied the formation of a weaker gluten network with an increasing frozen storage time. This led to a deterioration of final products’ quality, due to conformational changes in gluten. Figure 5B–F shows dough with KGM from 0.5% to 2.5% after 60 days of frozen storage. With 0.5% KGM, the gluten network increases slowly in aggregation. Granules are embedded in the gluten network of dough with KGM, especially dough with a high proportion of KGM. When KGM is added, smaller ice crystals formed during freezing, with less destruction of gluten. Since water was distributed more uniformly with high KGM, according to the results of LF-NMR, the association of starch and gluten protein was closer, improving the quality of the frozen dough.

### 3.3. Rheological and Tensile Properties of Frozen Dough

#### 3.3.1. Rheological Properties of Frozen Dough

**Figure 6:**
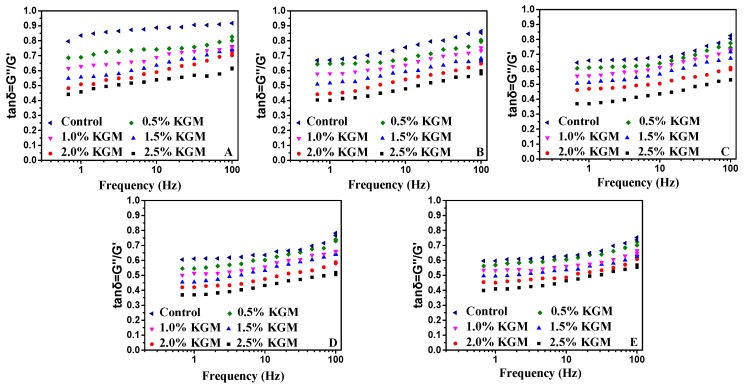
Effect of different proportions of KGM added on tanδ of frozen dough. 0 days frozen storage (**A**), 15 days frozen storage (**B**), 30 days frozen storage (**C**), 45 days frozen storage (**D**), 60 days frozen storage (**E**).

Analyses of the rheological and tensile properties of frozen dough with different proportions of KGM were conducted by using a dynamic rheometer and texture analyzer. Figure 6 shows the *tanδ* values for various frozen dough, calculated by the loss modulus (*G″*) and storage modulus (*G′*) (Figure S3) and used to describe the content and degree of high polymer polymerization in a dough system [36]. Similar trends were observed for each dough sample and samples with *tanδ* values less than 1 exhibited weak gel dynamic rheological properties. As the proportion of KGM added increased, *tanδ* decreased, *tanδ* decreased, and a higher content and polymerization degree were found. This result was inconsistent with Angioloni et al. [37], who reported the assessment of dough viscoelastic behavior with no yeast or additives, namely, that *tanδ* increased with storage time.

With the increased addition of KGM, *tanδ* decreased, indicating that KGM could protect gluten proteins and prohibit mechanical damage to the gluten network caused by freezing. Ribotta et al. [38] showed that gum guar could avoid the effect of frozen dough storage on the dynamic rheological parameters and improve the volume and texture of bread obtained from non-frozen and frozen dough. KGM and gum guar are polysaccharides used in in the baking industry, primarily to enhance the quality of the finished product. After 60 days with the addition of 2.5% KGM, samples had the lowest tanδ value in the control group without KGM addition, demonstrating that KGM has thickening, gelatinization, and other good properties and, as a result, that KGM solutions will become sols with the characteristics of pseudoplastic fluids [39]. It can thus be concluded that KGM slows the speed of deterioration of frozen dough.

From Figure 6A–E, the *tanδ* value of dough samples with 2.5% KGM added with freezing was the lowest among samples, with a storage time of up to 60 days. Samples without KGM showed values greater than 0.8, while the *tanδ* value of fresh samples was approximately 0.6. With longer freeze times, *tanδ* increased. This phenomenon was mainly caused by ice crystals in the dough, which destroyed the gluten structure during freeze-thaw cycles [40,41]. When the temperature increased, ice crystals were dissolved and dispersed through the gap between gluten proteins in the dough system, leading to increased gluten mobility. When the temperature dropped, ice crystals grew more frequently and their size increased, followed by recrystallization. In addition, the recrystallization degree directly depended on the temperature fluctuation. A greater temperature fluctuation [42] was correlated with the degree of recrystallization, which encouraged an increase in the size of ice crystal particles and rapidly decreased their quantity. In addition, ice crystal growth induced water redistribution, leading to reduced gluten cross-linking and the destruction of the frozen food structure. The thawed portion lost elasticity and influenced the final quality of flour products. Indeed, other authors have shown other effects of freezing, such as modification of starch properties and changes in proteins, which also influence the rheological properties of frozen dough [28].

#### 3.3.2. Tensile Properties of Frozen Dough

**Figure 7:**
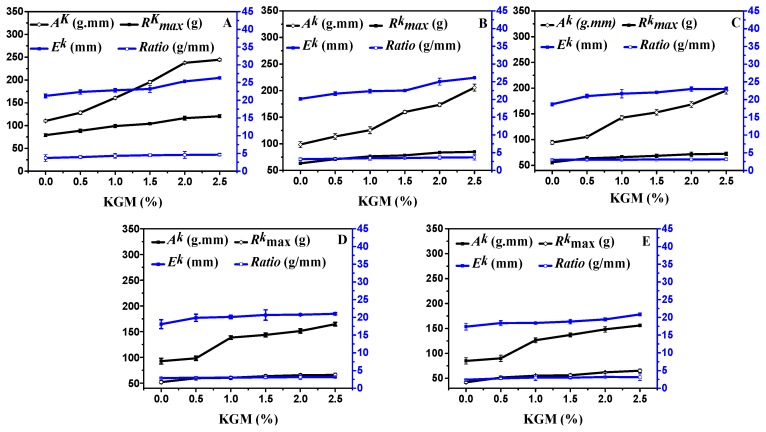
Tensile properties of frozen dough with different proportions of KGM added for 0 days frozen storage (**A**); 15 days frozen storage (**B**); 30 days frozen storage (**C**); 45 days frozen storage (**D**); and 60 days frozen storage (**E**).

The tensile maximum resistance (*R_k_^max^*), extensibility (*E^k^*), extension area (*A^k^*), and stretch ratio are the four main parameters that were significantly influenced by the proportion of KGM and frozen storage time and are exhibited in Figure 7. In fresh dough samples, when the storage time was extended to 60 days, the maximum resistance, extensibility, and extension area decreased by 37.24%, 5.84%, and 25.22%, respectively, and the stretch ratio slightly decreased. While three variables were increased at each storage time with KGM, especially at 0 day, when the storage time reached 60 days, three variables increased relatively less than those in fresh samples, and this phenomenon was affected by the structural components of the dough and protein and may have also been drastically altered by the recrystallization process. Yi and Kerr [43] showed that the bread quality from frozen dough depended on the rate of freezing, temperature, and length of time stored. Faster freezing and lower storage temperatures promote less damage to the gluten network, which could help retain the elastic properties of the dough. Longer frozen storage periods damaged both the gluten structure and yeast viability. This result was in accordance with the mechanical analyses. As a polysaccharide, KGM has good intrinsic viscosity and forms a gel with physical agglomeration. With 2.5% KGM, the dough’s tensile maximum resistance, extensibility, and extension area reached 64.22%, 21.43%, and 114.15%, respectively. Thus, the polysaccharide could not only restrain the reduction of the viscoelasticity of frozen dough, but also protect its structure [2,44].

### 3.4. Correlations Between Mechanical Characteristics and Structure Parameters

To further reveal the interaction mechanism of KGM and the gluten network in frozen dough, the study measured the relations between the mechanical characteristics and structure parameters. First, we tested how the structural parameters (*SH*_F_, *SS*, *β*-sheet, *T*_22,_ *T*_23,_ Δ*H*, and *T*_p_) related with the results of the mechanical properties (maximum resistance, extensibility, extension area, stretch ratio), which were obtained by experimental analyses. The correlation coefficients between the mechanical characteristics and structural parameters are shown in Table 3 (*r*^2^ > 0.6). Based on the mechanical characteristics, the parameters of maximum resistance, extensibility, extension area, and stretch ratio were linearly correlated with all of the structure parameters. Disulfide bonds play an important role in the formation of the three-dimensional structure of protein molecules. The SHF content was reduced with the increasing amount of KGM added, while SS increased, improving the rheological and tensile properties of frozen dough. The free water also showed greater liquidity, which may cause dough elasticity to decrease, thus affecting rheological and tensile properties.

All of the tensile properties, including the maximum resistance, extensibility, extension area, and stretch ratio, were linearly correlated with the structural parameters. It confirmed that KGM could improve the quality of frozen dough by converting free sulfhydryl bonds to disulfide bonds and increasing the *β*-sheet. The main factors, which can maintain the structural stability of gluten proteins, are hydrogen bonds, disulfide bonds, hydrophobic interaction, and covalent bonds. Disulfide bonds play an important role in maintaining the secondary structure. Moreover, enthalpy changes reflected the hydrophobicity and hydrophilicity of gluten protein molecules and the aggregation of protein molecules. Table 3 shows that the tensile properties had a close relationship with the enthalpy changes and *T*_p_. Therefore, KGM improved the formation of disulfide bonds and the *β*-sheet and enhanced the thermal stability of the system, leading to greater stability and crosslinking of the gluten network. Bound water also affected the tensile properties remarkably. Damages caused to gluten proteins by ice crystals may be the main reason for the deterioration of frozen dough or flour products. Kim et al. [2] found that ice crystals weakened the gluten network and extended the wake time of dough. Wang et al. [21] discovered that glutenin macropolymer depolymerization occurred during the frozen storage and led to major variations of free sulfhydryl. Comprehensive analysis and judgment showed that the primary reason for the degradation of the frozen dough quality was ice crystal formation and recrystallization, while control of ice crystal formation and recrystallization can maintain and enhance the quality of dough. During the formation of a gluten network, the addition of KGM could increase the content of weakly bound water and reduce the content of free water. Therefore, with the addition of KGM, the quality of frozen dough properties was improved and approached or reached the level of traditional flour products.

## 4. Conclusions

The protective effect of various amounts of KGM on the structural and physicochemical variations of gluten proteins/dough at different periods of frozen storage is evaluated in the present study. With the increase of frozen time, the *M*_w_ and the free sulfhydryl content of gluten proteins and the *R_k_^max^*, *E^k^*, and *A^k^* of frozen dough decreased, whereas the *β*-sheet and random coil contents of gluten proteins and the *tanδ* of frozen dough increased, with or without KGM addition. The addition of KGM could alleviate the variations of structural properties of gluten proteins and the alterations of rheological and tensile properties of frozen dough. Additionally, KGM interacted with gluten proteins and bound water in their structures, as indicated in the *T*_2_ distribution, SEM, and DSC results and the frozen dough with 2.5% KGM added showed the strongest water binding capacity, showed the fewest cracks and holes caused by ice crystals, and had the highest thermal stability. The correlations between mechanical characteristics and structure parameters further indicated that KGM could not only stabilize the structures of gluten proteins, but also bound free water to form a more stable complex and avoid the recrystallization of water, thereby protecting the variations in the rheological and tensile properties of frozen dough. Therefore, it can be inferred that the addition of KGM in frozen dough may make the final products obtain acceptable texture and sensory characteristics.

## Figures and Tables

**Table 1:** Effect of different proportions of KGM added to the molecular weight of frozen dough.

Storage Time (d)	KGM (%)	Protein Molecular Weight				
		*M*_w_ (Da)	Peak 1 Area (%)	Peak 2 Area (%)	Peak 3 Area (%)	Peak 4 Area (%)
0	0	1.98 × 10^5^	23.35 ± 0.27 ^f^	48.59 ± 0.37 ^f^	18.52 ± 0.11 ^a^	9.54 ± 0.03 ^a^
	0.5	2.10 × 10^5^	25.23 ± 0.09 ^e^	49.56 ± 0.11 ^e^	18.12 ± 0.13 ^b^	7.09 ± 0.04 ^b^
	1.0	2.16 × 10^5^	26.13 ± 0.11 ^d^	50.73 ± 0.12 ^cd^	17.62 ± 0.31 ^c^	5.52 ± 0.04 ^c^
	1.5	2.18 × 10^5^	27.62 ± 0.08 ^c^	51.08 ± 0.45 ^bc^	17.22 ± 0.04 ^d^	4.08 ± 0.04 ^d^
	2.0	2.20 × 10^5^	29.13 ± 0.16 ^ab^	51.20 ± 0.22 ^b^	17.03 ± 0.16 ^de^	2.64 ± 0.03 ^e^
	2.5	2.25 × 10^5^	29.24 ± 0.14 ^a^	51.83 ± 0.31 ^a^	16.63 ± 0.03 ^f^	2.30 ± 0.06 ^f^
15	0	1.87 × 10^5^	22.12 ± 0.11 ^e^	45.36 ± 0.06 ^d^	18.94 ± 0.10 ^d^	13.58 ± 0.03 ^c^
	0.5	2.05 × 10^5^	22.61 ± 0.17 ^d^	45.61 ± 0.09 ^c^	18.66 ± 0.10 ^a^	12.84 ± 0.15 ^a^
	1.0	2.10 × 10^5^	23.17 ± 0.09 ^d^	46.57 ± 0.15 ^c^	18.47 ± 0.06 ^b^	11.79 ± 0.10 ^b^
	1.5	2.13 × 10^5^	23.52 ± 0.06 ^c^	47.69 ± 0.13 ^c^	18.36 ± 0.15 ^b^	10.43 ± 0.04 ^d^
	2.0	2.16 × 10^5^	24.63 ± 0.03 ^b^	48.70 ± 0.10 ^b^	17.95 ± 0.02 ^c^	8.72 ± 0.06 ^e^
	2.5	2.22 × 10^5^	25.39 ± 0.01 ^a^	49.26 ± 0.16 ^a^	17.75 ± 0.06 ^c^	7.60 ± 0.07 ^f^
30	0	1.65 × 10^5^	21.14 ± 0.10 ^e^	44.71 ± 0.13 ^d^	19.29 ± 0.08 ^e^	14.86 ± 0.07 ^a^
	0.5	2.00 × 10^5^	21.53 ± 0.04 ^d^	45.07 ± 0.10 ^a^	19.16 ± 0.10 ^d^	14.24 ± 0.04 ^b^
	1.0	2.06 × 10^5^	21.83 ± 0.13 ^c^	45.20 ± 0.12 ^b^	18.89 ± 0.17 ^c^	14.08 ± 0.05 ^c^
	1.5	2.10 × 10^5^	22.17 ± 0.08 ^c^	45.98 ± 0.09 ^b^	18.89 ± 0.14 ^b^	12.96 ± 0.06 ^d^
	2.0	2.11 × 10^5^	23.40 ± 0.07 ^b^	46.74 ± 0.17 ^c^	18.62 ± 0.08 ^b^	11.78 ± 0.09 ^e^
	2.5	2.14 × 10^5^	24.94 ± 0.14 ^a^	47.46 ± 0.17 ^c^	18.35 ± 0.05 ^a^	9.26 ± 0.02 ^f^
45	0	1.51 × 10^5^	21.09 ± 0.08 ^d^	41.14 ± 0.01 ^e^	19.93 ± 0.12 ^f^	17.84 ± 0.17 ^a^
	0.5	1.84 × 10^5^	21.46 ± 0.04 ^d^	41.36 ± 0.06 ^d^	19.63 ± 0.10 ^e^	16.95 ± 0.01 ^b^
	1.0	2.01 × 10^5^	21.17 ± 0.12 ^c^	43.62 ± 0.11 ^c^	19.01 ± 0.04 ^d^	16.22 ± 0.13 ^c^
	1.5	2.07 × 10^5^	22.76 ± 0.09 ^b^	44.60 ± 0.16 ^c^	18.99 ± 0.06 ^c^	13.65 ± 0.10 ^d^
	2.0	2.08 × 10^5^	22.93 ± 0.05 ^b^	46.56 ± 0.01 ^b^	18.65 ± 0.10 ^b^	11.86 ± 0.01 ^e^
	2.5	2.11 × 10^5^	24.58 ± 0.12 ^a^	46.75 ± 0.15 ^a^	18.33 ± 0.12 ^a^	11.34 ± 0.05 ^f^
60	0	1.39 × 10^5^	20.54 ± 0.11 ^e^	41.05 ± 0.10 ^f^	20.06 ± 0.10 ^f^	18.35 ± 0.16 ^a^
	0.5	1.75 × 10^5^	20.83 ± 0.05 ^d^	41.80 ± 0.14 ^e^	19.78 ± 0.15 ^e^	17.59 ± 0.10 ^b^
	1.0	2.00 × 10^5^	21.46 ± 0.12 ^c^	42.36 ± 0.14 ^d^	19.37 ± 0.19 ^d^	16.81 ± 0.12 ^c^
	1.5	2.06 × 10^5^	21.62 ± 0.05 ^c^	42.97 ± 0.19 ^c^	19.03 ± 0.12 ^c^	16.38 ± 0.04 ^d^
	2.0	2.06 × 10^5^	22.15 ± 0.42 ^b^	43.50 ± 0.15 ^b^	18.98 ± 0.14 ^b^	15.37 ± 0.10 ^e^
	2.5	2.08 × 10^5^	23.24 ± 0.11 ^a^	44.39 ± 0.25 ^a^	18.29 ± 0.18 ^a^	14.08 ± 0.09 ^f^

Data are expressed as the mean value (*n* = 3) ± standard deviation. Means at the same storage time with different superscript letters in the same column indicate a significant difference (*p* < 0.05).

**Table 2:** Effect of different proportions of KGM added on T2 distribution and the thermal parameter of frozen dough.

Storage Time (d)	KGM (%)	*T*_2_ Distribution Area (%)			Thermal Parameter	
		*T*_21_	*T*_22_	*T*_23_	*T*_p_ (°C)	∆*H*/(J/g)
0	0	10.76 ± 0.12 ^d^	80.05 ± 0.56 ^c^	9.19 ± 0.12 ^a^	54.74 ± 2.35 ^d^	114.45 ± 6.54 ^f^
	0.5	11.04 ± 0.16 ^c^	81.89 ± 0.35 ^b^	6.47 ± 0.16 ^b^	62.70 ± 1.56 ^c^	123.49 ± 3.31 ^e^
	1.0	11.18 ± 0.43 ^c^	81.99 ± 0.45 ^b^	5.83 ± 0.09 ^c^	67.30 ± 1.30 ^b^	129.07 ± 1.33 ^d^
	1.5	11.29 ± 0.48 ^bc^	82.80 ± 0.79 ^b^	4.91 ± 0.18 ^d^	67.43 ± 2.13 ^bc^	153.92 ± 4.21 ^bc^
	2.0	11.74 ± 0.08 ^b^	84.61 ± 0.35 ^a^	3.65 ± 0.23 ^e^	69.54 ± 1.16 ^b^	156.22 ± 1.64 ^b^
	2.5	12.05 ± 0.14 ^a^	85.55 ± 1.79 ^a^	2.40 ± 0.44 ^f^	72.75 ± 1.25 ^a^	160.32 ± 2.35 ^a^
15	0	10.17 ± 0.02 ^a^	78.32 ± 0.06 ^a^	11.51 ± 0.03 ^a^	56.65 ± 1.25 ^f^	112.10 ± 3.56 ^f^
	0.5	10.23 ± 0.03 ^b^	81.03 ± 0.43 ^b^	8.74 ± 0.01 ^b^	63.58 ± 2.21 ^de^	119.04 ± 2.54 ^de^
	1.0	10.50 ± 0.08 ^c^	81.24 ± 0.03 ^b^	8.26 ± 0.03 ^c^	67.94 ± 2.58 ^cd^	120.47 ± 1.58 ^cd^
	1.5	10.68 ± 0.12 ^cd^	81.84 ± 0.06 ^c^	7.48 ± 0.21 ^d^	68.16 ± 2.34 ^bc^	125.93 ± 4.23 ^bc^
	2.0	10.73 ± 0.21 ^cd^	82.22 ± 0.43 ^cd^	7.05 ± 0.37 ^e^	69.98 ± 1.24 ^b^	128.70 ± 2.58 ^ab^
	2.5	10.91 ± 0.10 ^cd^	82.37 ± 0.21 ^de^	6.72 ± 0.09 ^f^	73.53 ± 1.99 ^a^	130.86 ± 4.54 ^a^
30	0	9.62 ± 0.32 ^a^	78.13 ± 0.21 ^a^	12.25 ± 0.25 ^a^	58.42 ± 2.34 ^e^	106.64 ± 3.45 ^e^
	0.5	9.82 ± 0.05 ^b^	80.98 ± 0.21 ^b^	9.20 ± 0.41 ^b^	65.72 ± 2.58 ^cd^	110.24 ± 4.12 ^cd^
	1.0	10.36 ± 0.11 ^c^	81.01 ± 0.31 ^bc^	8.63 ± 0.65 ^cd^	70.80 ± 3.16 ^bc^	111.85 ± 3.87 ^cd^
	1.5	10.42 ± 0.21 ^cd^	81.54 ± 0.33 ^bc^	8.04 ± 0.22 ^cd^	71.05 ± 2.87 ^ab^	116.41 ± 2.56 ^c^
	2.0	10.53 ± 0.11 ^cd^	81.77 ± 0.12 ^cd^	7.70 ± 0.10 ^e^	74.23 ± 3.01 ^ab^	122.04 ± 2.88 ^ab^
	2.5	10.83 ± 0.21 ^cd^	81.78 ± 0.44 ^cde^	7.39 ± 0.43 ^f^	76.77 ± 1.58 ^a^	125.93 ± 3.66 ^a^
45	0	9.43 ± 0.28 ^a^	77.96 ± 0.31 ^a^	12.61 ± 0.36 ^a^	59.66 ± 1.05 ^e^	91.40 ± 3.54 ^ef^
	0.5	9.63 ± 0.13 ^b^	80.41 ± 0.06 ^b^	9.96 ± 0.22 ^b^	67.38 ± 2.71 ^cd^	94.56 ± 3.68 ^e^
	1.0	9.88 ± 0.25 ^bc^	80.96 ± 0.45 ^c^	9.16 ± 0.37 ^cd^	71.21 ± 2.11 ^cd^	104.91 ± 3.44 ^d^
	1.5	10.06 ± 0.38 ^bc^	81.12 ± 0.43 ^d^	8.82 ± 0.06 ^cd^	73.48 ± 3.25 ^bc^	110.36 ± 3.25 ^c^
	2.0	10.14 ± 0.09 ^bc^	81.32 ± 0.22 ^de^	8.54 ± 0.23 ^ef^	76.02 ± 3.66 ^ab^	116.78 ± 2.58 ^ab^
	2.5	10.60 ± 0.23 ^bcd^	81.54 ± 0.56 ^ef^	7.86 ± 0.41 ^e^	79.43 ± 3.54 ^a^	117.23 ± 3.42 ^a^
60	0	9.20 ± 0.01 ^a^	77.65 ± 0.41 ^a^	13.15 ± 0.24 ^a^	62.62 ± 1.22 ^f^	82.78 ± 2.53 ^ef^
	0.5	9.46 ± 0.26 ^b^	79.20 ± 0.26 ^b^	11.34 ± 0.21 ^b^	69.02 ± 3.47 ^de^	85.14 ± 2.68 ^de^
	1.0	9.50 ± 0.33 ^bc^	80.42 ± 0.22 ^bc^	10.08 ± 0.44 ^c^	73.99 ± 2.55 ^cd^	87.10 ± 2.54 ^d^
	1.5	9.91 ± 0.02 ^bc^	80.54 ± 0.11 ^bc^	9.55 ± 0.09 ^d^	75.43 ± 3.68 ^bc^	93.96 ± 3.65 ^bc^
	2.0	9.94 ± 0.12 ^bc^	80.88 ± 0.03 ^d^	9.18 ± 0.15 ^ef^	76.28 ± 3.87 ^ab^	98.75 ± 3.87 ^ab^
	2.5	10.09 ± 0.26 ^bc^	80.96 ± 0.09 ^d^	8.95 ± 0.22 ^ef^	80.23 ± 2.44 ^a^	104.91 ± 4.57 ^a^

Data are expressed as the mean value (n = 3) ± standard deviation. Means at the same storage time with different superscript letters in the same column indicate a significant difference (*p* < 0.05).

**Table 3:** The correlations between tensile characteristics and structure parameters.

	*M*_w_	*SH*_F_	*SS*	*β*-Sheet	*T*_22_	*T*_23_	△*H*	*T*_p_
***R_k_^max^* (g)**	0.899 **	−0.986 **	0.996 **	0.990 **	0.925 **	−0.951 **	0.960 **	0.962 **
***E^k^* (mm)**	0.785 **	−0.913 **	0.970 **	0.951 **	0.825 **	−0.862 **	0.895 **	0.973 **
***A^k^* (g.mm)**	0.855 **	−0.966 **	0.990 **	0.980 **	0.883 **	−0.919 **	0.923 **	0.923 **
**Stretch ratio**	0.659 **	−0.914 **	0.947 **	0.917 *	0.784 **	−0.812 **	0.811 **	0.854 **

** Correlation is significant at a level of 0.01 (2-tailed); * Correlation is significant at a level of 0.05 (2-tailed).

## Supplementary Materials

The following are available online at https://www.mdpi.com/2073-4360/11/5/794/s1, Figure S1: FTIR spectra of gluten samples with different proportions of KGM added on frozen dough, Figure S2: DSC thermograms of gluten samples with different proportions of KGM added on frozen dough, Figure S3: Rheological properties of frozen dough with different KGM added. G′ (A) and G″ (B) for frozen dough without KGM added; G′ (C) and G″ (D) for frozen dough with 0.5% KGM added; G′ (E) and G″ (F) for frozen dough with 1.0% KGM added; G′ (G) and G″ (H) for frozen dough with 1.5% KGM added; G′ (I) and G″ (J) for frozen dough with 2.0% KGM added; G′ (K) and G″ (L) for frozen dough with 2.5% KGM added.
