# Supplementary material for: Influence of Konjac Glucomannan and Frozen Storage on Rheological and Tensile Properties of Frozen Dough
Source: Polymers (Basel). 2019 May 2;11(5):794. doi: 10.3390/polym11050794 (PMC6572217; doi:10.3390/polym11050794)
Supplement: Supplementary file 1 [file polymers-11-00794-s001.pdf]

Supplementary Materials

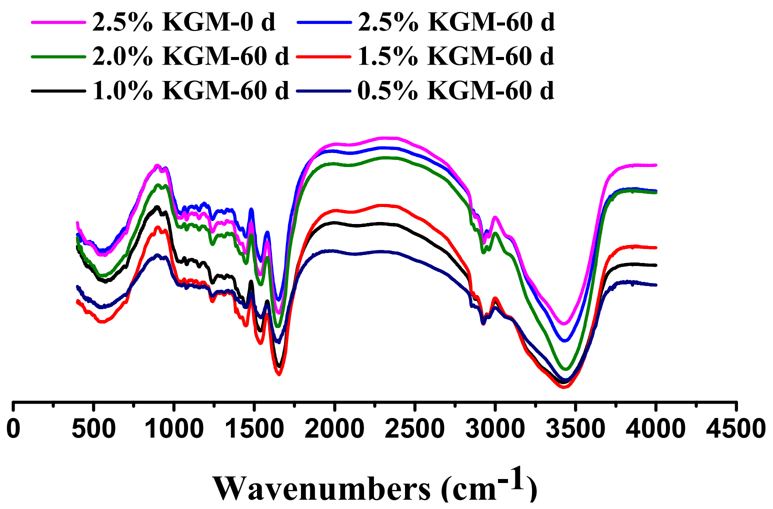

Figure S1 FTIR spectra of gluten with different proportions of KGM added on frozen dough.

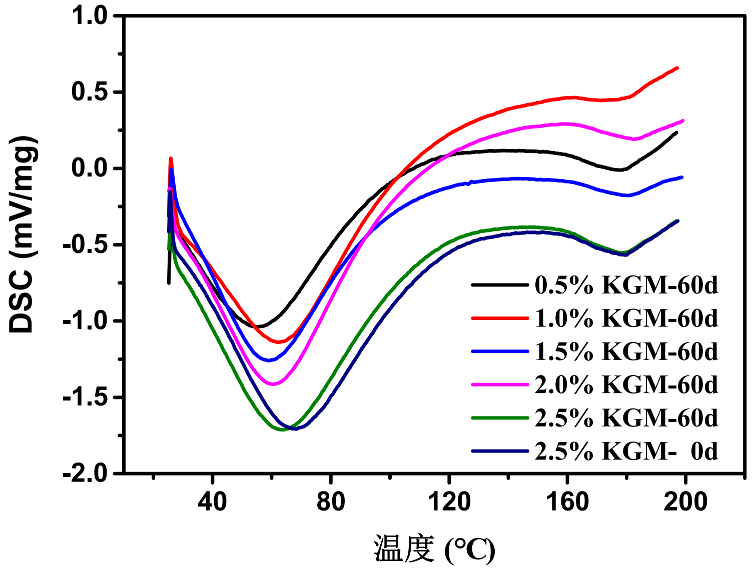

Figure S2 DSC thermogram of gluten with different proportions of KGM added on frozen dough.

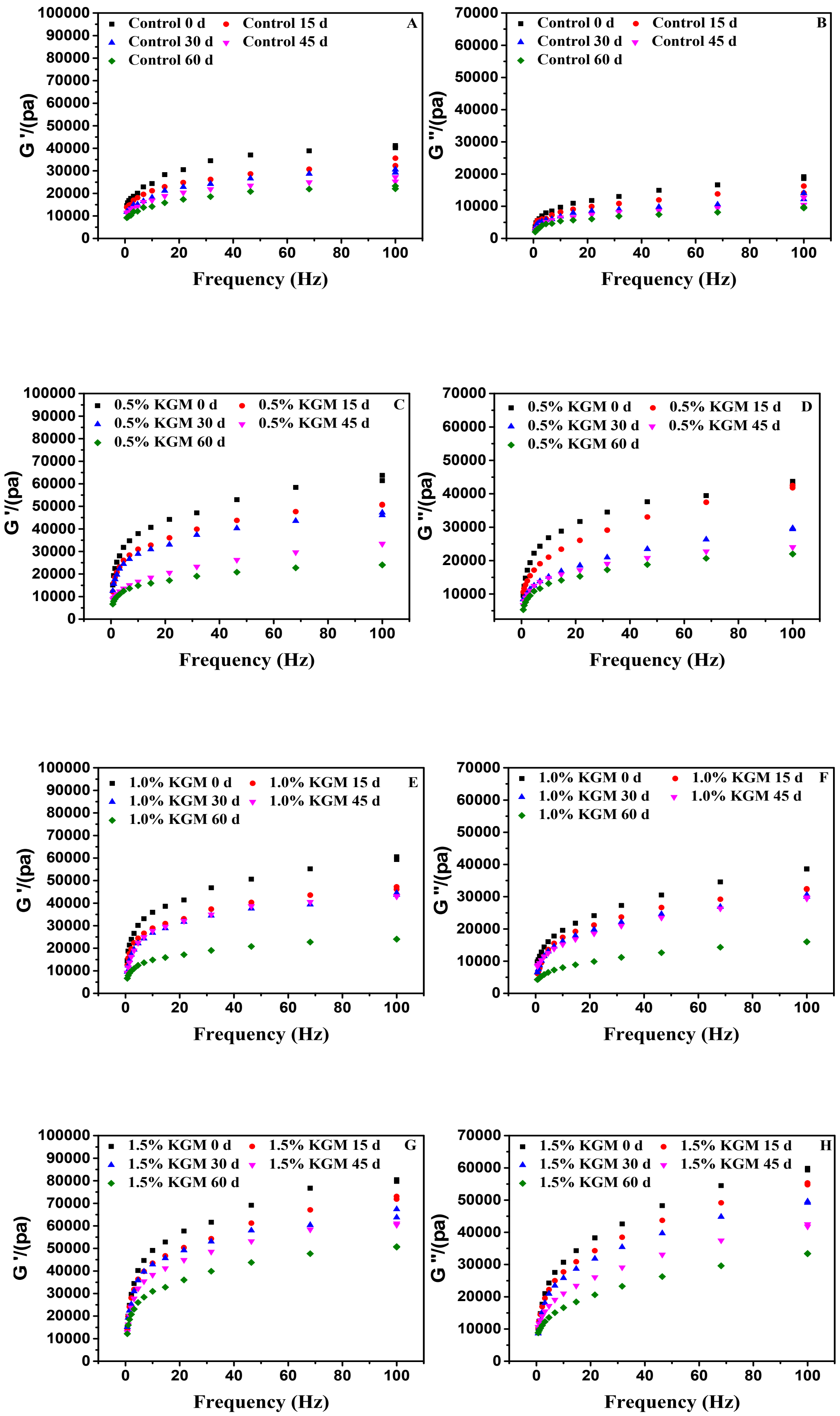
A
Control 0 d
Control 15 d
Control 30 d
Control 45 d
Control 60 d
G '/(pa)
Frequency (Hz)
B
Control 0 d
Control 15 d
Control 30 d
Control 45 d
Control 60 d
G "/(pa)
Frequency (Hz)
C
0.5% KGM 0 d
0.5% KGM 15 d
0.5% KGM 30 d
0.5% KGM 45 d
0.5% KGM 60 d
G '/(pa)
Frequency (Hz)
D
0.5% KGM 0 d
0.5% KGM 15 d
0.5% KGM 30 d
0.5% KGM 45 d
0.5% KGM 60 d
G "/(pa)
Frequency (Hz)
E
1.0% KGM 0 d
1.0% KGM 15 d
1.0% KGM 30 d
1.0% KGM 45 d
1.0% KGM 60 d
G '/(pa)
Frequency (Hz)
F
1.0% KGM 0 d
1.0% KGM 15 d
1.0% KGM 30 d
1.0% KGM 45 d
1.0% KGM 60 d
G "/(pa)
Frequency (Hz)
G
1.5% KGM 0 d
1.5% KGM 15 d
1.5% KGM 30 d
1.5% KGM 45 d
1.5% KGM 60 d
G '/(pa)
Frequency (Hz)
H
1.5% KGM 0 d
1.5% KGM 15 d
1.5% KGM 30 d
1.5% KGM 45 d
1.5% KGM 60 d
G "/(pa)
Frequency (Hz)

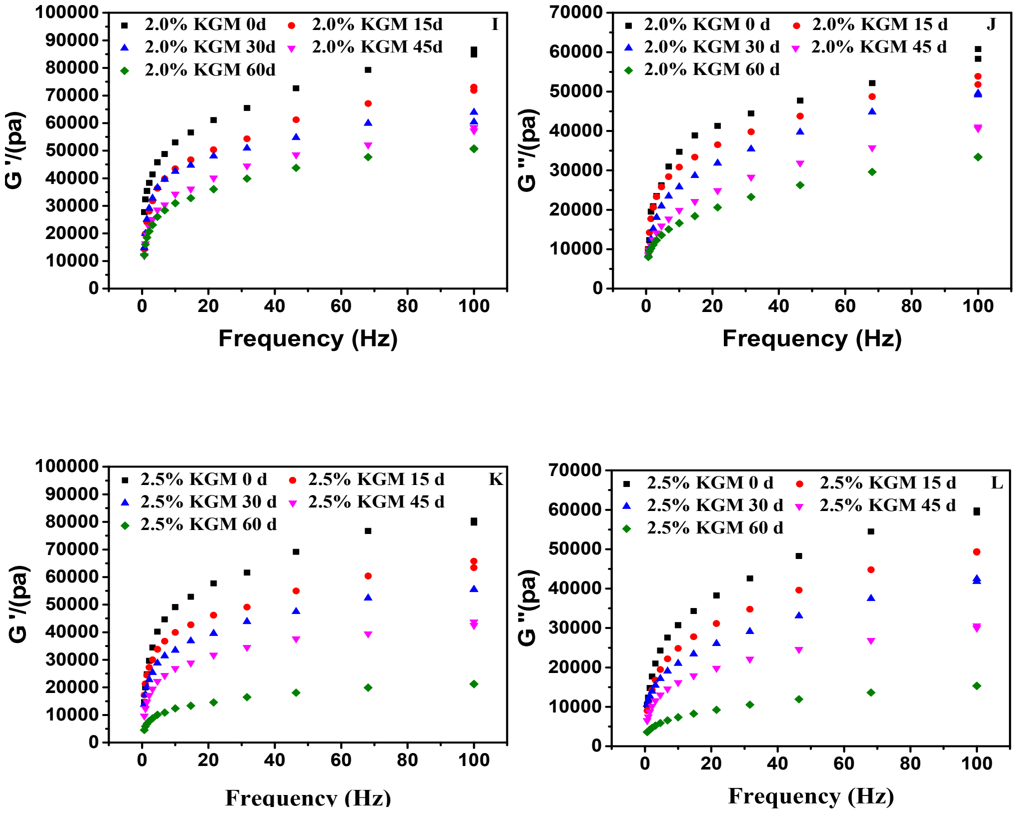

Figure S3 Rheological properties of frozen dough with different KGM added. G' (A) and G'' (B) for frozen dough without KGM added; G' (C) and G'' (D) for frozen dough with 0.5% KGM added; G' (E) and G'' (F) for frozen dough with 1.0% KGM added; G' (G) and G'' (H) for frozen dough with 1.5% KGM added; G' (I) and G'' (J) for frozen dough with 2.0% KGM added; G' (K) and G'' (L) for frozen dough with 2.5% KGM added.
